# Effects of reverse osmosis membrane replacement of pure water system on clinical chemistry and immunoassay in clinical laboratory

**DOI:** 10.11613/BM.2024.010705

**Published:** 2024-02-15

**Authors:** Shaocong Liang, Huaxian Wu, Jiayi Zhao, Xuanjie Guo, Yongjie Qiang, Xin Zhao, Meng Lan, Chongquan Zhao, Dongxin Zhang

**Affiliations:** 1 Department of Laboratory Medicine, Zhujiang Hospital, Southern Medical University, Guangzhou, China; 2 Department of Clinical Laboratory, Affiliated Maternal & Child Health Hospital, Southern Medical University, Foshan, China

**Keywords:** water purification, glycerol, clinical chemistry tests, immunoassay, triglycerides

## Abstract

**Introduction:**

Reverse osmosis (RO) membrane, key component of water-purifying equipment, is often stored in protection fluid containing substances such as glycerol, which may contaminate the water at replacement. This study aims to explore the effects of RO membrane replacement on clinical chemistry and immunoassay, particularly triglyceride (TG), providing reference for managing test interference caused by RO membrane replacement.

**Materials and methods:**

The RO membrane of water-purifying equipment A, which provided water to C16000 biochemistry analyzer (Abbott Laboratories, Abbott Park, USA) and E801 electrochemiluminescence analyzer (Roche, Basel, Switzerland), was replaced. Water resistivity was recorded, and quality control (QC) tests were performed on C16000 and E801. Moreover, TG was measured in 29 of selected serum samples on C16000 at 0.5h and 10.5h after RO membrane replacement and on reference biochemistry analyzer BS2000M (Mindray Biomedical Electronics Co., Shenzhen, China), which was connected to water-purifying equipment B without RO membrane replacement. Finally, blank, calibrator 1 and calibrator 2 of TG reagent were measured on C16000 before and at 0.5h, 2.5h and 10.5h after RO membrane replacement. All statistical analyses of data were done using GraphPad Prism (GraphPad Software Inc., San Diego, USA), and a value of P < 0.05 was considered statistically significant.

**Results:**

After RO membrane replacement, all QC results of clinical chemistry and immune tests passed except TG that showed positive bias of 536% and 371% at two levels, respectively. Moreover, TG results of the same serum samples were significantly higher at 0.5h than 10.5h after RO membrane replacement. Meanwhile, there was worse agreement and correlation of TG results between C16000 and BS2000M at 0.5h than 10.5h after replacement. Furthermore, the absorbance of TG blank, calibrator 1 and calibrator 2 was significantly higher at 0.5h and 2.5h after replacement than before replacement, and the absorbance gradually returned to normal value at 10.5h after replacement.

**Conclusions:**

Replacement of RO membrane could cause significant interference to TG test while have no effects on other laboratory tests performed in the study, which may be due to glycerol contamination. Our data provides important reference for management of test interference caused by RO membrane replacement. Clinical laboratory should observe the effects of RO membrane replacement on laboratory tests through both water quality monitoring and QC detection.

## Graphical abstract

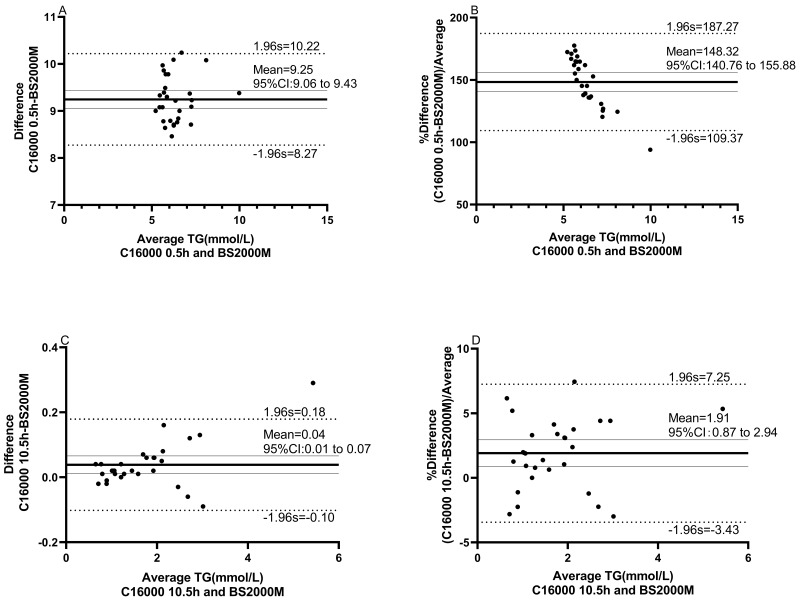

## Highlights

• The effects of reverse osmosis membrane replacement on clinical chemistry and immunoassay were showed

• Reverse osmosis membrane replacement interferes triglyceride test

• Triglyceride interference by reverse osmosis membrane replacement may be due to glycerol carryover

• The data provide reference for management of triglyceride interference by reverse osmosis membrane replacement

## Introduction

Pure water is extensively used in clinical laboratory since it has a wide range of applications, including dilution of samples and reagents, cleaning of sample probe, reagent probe, reaction cuvettes and instrument pipelines as well as preparation of calibrators, controls and reagents. Moreover, it can even participate in testing processes as substrate (*1*). Therefore, high quality of pure water is essential for laboratory testing (*2*). False test results due to poor water quality can have serious impact on patients, since clinical physicians increasingly rely on test results for clinical diagnosis and treatment (*3*). With the development of water-purifying technology, clinical laboratory generally has stable access to pure water that meets required standard. Unfortunately, unexpected errors can still occur at any phases of laboratory testing (*4*).

Water-purifying technologies includes distillation, reverse osmosis (RO), activated carbon adsorption, ion exchange, *etc*. (*2*). Many clinical laboratories adopt RO water-purifying equipment. The main principle of water-purifying equipment in our laboratory is the combination of RO and electrodeionization technology (*5*, *6*). Reverse osmosis membrane is an artificial semi-permeable microporous membrane that is the core component of RO water-purifying equipment. The in-put water is under the pressure which is higher than the opposing osmotic pressure generated by the concentration gradient across the RO membrane (*7*). Reverse osmosis membrane can optionally remove viruses, bacteria, solid soluble, calcium and magnesium ions, *etc*. to achieve the purpose of water purification. Laboratories usually confirm whether the water quality meets the requirements by recording the resistivity of pure water, conducting microbial cultivation of pure water, *etc*.

As consumable material, RO membrane needs to be replaced regularly to ensure best water-purifying effects. Before replacement, RO membrane is often stored in protection fluid containing glycerol, which may contaminate the pure water at replacement. Glycerol is also used as a well antifreeze of RO membrane, and even as lubricant during installation. Therefore, newly replaced RO membrane brings the risk of glycerol contamination in pure water. As known, TG is detected measured by employing GPO-PAP-enzymatic colorimetric method in clinical laboratory, based on the sequence of reactions proposed by Fossati *et al.* and McGowan *et al.* (*8*, *9*). During test processes, TG in sample is firstly hydrolyzed into free fatty acids and glycerol, and the latter further participates in following reactions. Thus, TG concentrations in the sample are directly proportional to the glycerol produced from above reaction. Previous study reported that free glycerol in the sample can have positive interference on the detection of TG (*10*). However, there are currently no studies on the effects of potential contamination of pure water on laboratory testing results caused by RO membrane replacement. Thus, the effects of RO membrane replacement on laboratory testing should be studied systematically since the quality of pure water is key to testing processes.

The purpose of the present study was to investigate the effects of RO membrane replacement on clinical chemistry tests and immunoassay, particularly TG, providing important reference for eliminating the effects of RO membrane replacement on laboratory tests.

## Materials and methods

### Materials

Twenty-nine of serum samples were randomly selected from blood samples of patients undergoing clinical laboratory testing at Department of Laboratory Medicine, Zhujiang Hospital, Southern Medical University, Guangzhou, China. Blood samples were taken from patients using vacuum blood collection tube (tube with coagulant; Improve Medical Instruments, Guangzhou, China). Blood samples were centrifuged at 3300xg for 10 minutes for separating serum used for clinical laboratory testing as well as our study.

### Instruments

Both water-purifying system A (HJJ-ROEDI500, Jingyuan, Guangzhou, China) and water-purifying system B (HT-RO-0.5m3, Runji, Guangzhou, China) were equipped with RO membrane (HALLOWWAY, Suzhou, China). Clinical biochemical tests were performed on C16000 biochemistry analyzer (Abbott Laboratories, Abbott Park, USA)-and BS2000M biochemistry analyzer (Mindray Biomedical Electronics, Shenzhen, China). Clinical immunoassay was performed on E801 electrochemiluminescence analyzer (Roche, Basel, Switzerland). Both C16000 and E801 used pure water supplied by water-purifying equipment A with RO membrane replacement, while BS2000M used pure water supplied by water-purifying equipment B without RO membrane replacement. Here, C16000 and BS2000M were connected to the water supply with the continuous flow of water, while E801 was connected to the water supply of water-purifying equipment through a built-in buffer tank (volume = 10 L). The buffer tank soothes out sudden changes in water pressure and flow rate. Since E801 has 30 L/h of continuous water consumption requirement, the buffer tank only provides brief water supply sustaining E801 operation.

### Reagents

All of biochemical test reagents, including potassium (K), sodium (Na), chloride (Cl), calcium (Ca), creatinine (Crea), urea, glucose (Glu), total protein (TP), cholesterol (Chol), TG, alanine aminotransferase (ALT), aspartate aminotransferase (AST), alkaline phosphatase (ALP) and total bilirubin (TBIL) were from Abbott Laboratories (Abbott Park, USA) except albumin (Alb, Aidian Oy, Espoo, Finland). Calibrator for TG (lot number: 87719F01) was also from Abbott Laboratories (Abbott Park, USA). All clinical immunoassay reagents, including alpha-fetoprotein (AFP), carcinoembryonic antigen (CEA), carbohydrate antigen 19-9 (CA19-9), carbohydrate antigen 125 (CA125), carbohydrate antigen 15-3 (CA15-3), free triiodothyronine (fT3), free thyroxine (fT4) and thyroid-stimulating hormone (TSH) were from Roche (Basel, Switzerland). Reagent for TG test on BS2000M was from Mindray Biomedical Electronics Co. (Shenzhen, China). Quality controls (QCs) of all above biochemical items were measured by using Liquid Assayed Multiqual (Bio-Rad Laboratories, Hercules, USA). Quality controls of tumor markers were measured by using Lyphochek Tumor Marker Plus Control (Bio-Rad Laboratories, Hercules, USA). Quality controls of hormones were measured by using Lyphochek Immunoassay Plus Control (Bio-Rad Laboratories, Hercules, USA).

### Methods

This observational study was performed in March 2021 at Department of Laboratory Medicine, Zhujiang Hospital, Southern Medical University, Guangzhou, China.

In this study, the RO membrane of water-purifying equipment A was replaced due to the imminent expiration of three-year usage limit. Prior to replacement, the laboratory routinely recorded the resistivity of pure water produced by equipment A. After replacement, equipment A continuously produced and drained water, and the resistivity values were recorded again at 0.5h, 2.5h and 10.5h to confirm whether water-purifying equipment was operating normally. Resistivity value > 10 MΩ·cm indicated that the water quality met the requirements. Quality control testing of clinical chemistry and immunoassay was performed on C16000 and E801 before and within 0.5h after replacement to confirm that QC was under control. Clinical chemistry tests would be completed within 15 minutes, while immunoassays would take up to one hour.

Triglyceride was measured in 29 serum samples on C16000 and BS2000M within 0.5 hours after RO membrane replacement. Here, BS2000M served as the reference biochemistry analyzer, since it was connected to water-purifying equipment B without RO membrane replacement. At 10.5 hours after replacement, the final TG calibration mentioned below was completed. Then, TG of 29 serum samples was retested on C16000 to confirm the change of TG results after flushing RO system.

Triglyceride calibration on C16000 was performed at 0.5h after the replacement, at which time TG of QC and samples had already been tested. Then, calibration was performed again at 2.5h and 10.5h to analyze the change of absorbance values with replacement time. During each calibration process, the C16000 automatically performed three repeated measurements and recorded the absorbance values to generate the calibration curve. The absorbance values of blank, calibrator 1 and calibrator 2 from the last successful calibration before replacement were set as the reference. In the TG calibration, the blank determination refers to the measurement of pure water from water-purifying equipment, and the blank value is the final absorbance of pure water added to the cuvette by C16000 automatically as a sample for detection. This blank will be recorded in C16000 and deducted from subsequent absorbance measurements to eliminate its influence.

### Statistical analysis

All statistical analyses were done using GraphPad Prism version 8.0 (GraphPad Software, San Diego, USA). Quality control bias was calculated using the formula: bias = (value_after_ - value_before_) / value_before_. If the bias was ≤ 1/3 total allowable error (TEa) (TEa refers to the evaluation criterion for each clinical testing item in China’s External Quality Assessment), it passed, otherwise not. The Shapiro-Wilk test was used for assessment of data normality. Continuous variables with normal distribution were expressed as means ± standard deviation (SD), and comparisons were made using one-way ANOVA followed by Dunnett’s *post-hoc* test. Skewed data were expressed as median and interquartile range (IQR), and comparisons were made using Wilcoxon test. A value of P < 0.05 was considered significant. Triglyceride results of serum samples between two biochemical analyzers were analyzed by Passing-Bablok regression, Spearman’s correlation analysis and Bland-Altman plots.

## Results

### Changes of water resistivity of water-purifying equipment A before and after RO membrane replacement

**Figure 1:**
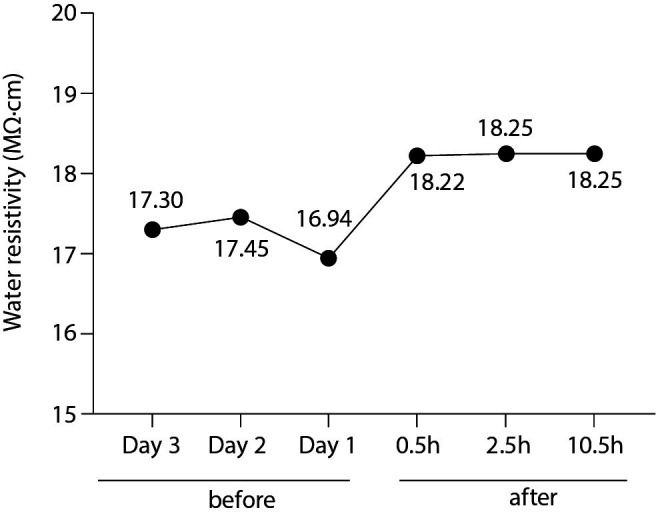
The changes of water resistivity of water-purifying equipment A before and after RO membrane replacement. The water resistivity of water-purifying equipment A was recorded three days before RO membrane replacement and at 0.5h, 2.5h and 10.5h after replacement. RO - reverse osmosis.

Figure 1 shows the changes of water resistivity at different time before and after RO membrane replacement. Water resistivity consistently met the required standard (> 10 MΩcm) before and after RO membrane replacement, although it increased after replacement.

### Comparison of QC results of clinical chemistry and immunoassay before and after RO membrane replacement

Table 1 and 2 shows the bias of QC results of clinical chemistry and immunoassay tests. Except for TG, the bias of all other test items was within 1/3 TEa and passed the comparison. The QC result of TG increased significantly after RO membrane replacement with the bias reaching 536% and 371% at two levels respectively.

**Table 1:** Comparison of quality control results of clinical biochemistry tests before and after the reverse osmosis membrane replacement

**Parameter**	**QC level**	**QC results** **before replacement**	**QC results** **after replacement**	**Bias** **(%)**	**1/3 TEa** **(%)**	**Conclusion**
K, mmol/L	1	3.8	3.8	0.79	2.0	Pass
	2	7.6	7.6	0.13	2.0	Pass
Na, mmol/L	1	140	141	1.2	1.3	Pass
	2	157	158	0.25	1.3	Pass
Cl, mmol/L	1	101	101	0.70	1.3	Pass
	2	123	123	- 0.16	1.3	Pass
Ca, mmol/L	1	2.43	2.47	1.7	1.7	Pass
	2	3.20	3.23	0.94	1.7	Pass
Crea, μmol/L	1	153	153	- 0.26	4.0	Pass
	2	586	585	- 0.19	4.0	Pass
Urea, mmol/L	1	14.0	13.8	- 1.0	2.7	Pass
	2	25.2	25.1	- 0.12	2.7	Pass
Glu, mmol/L	1	6.5	6.5	0.77	2.3	Pass
	2	20.6	20.3	- 1.5	2.3	Pass
TP, g/L	1	54	55	0.18	1.7	Pass
	2	73	72	- 0.83	1.7	Pass
Alb, g/L	1	38	37	- 1.9	2.0	Pass
	2	47	46	- 1.5	2.0	Pass
Chol, mmol/L	1	4.5	4.6	1.6	3.0	Pass
	2	6.7	6.7	0.30	3.0	Pass
TG, mmol/L	1	1.5	9.7	536	4.7	Not pass
	2	2.3	10.9	371	4.7	Not pass
ALT, IU/L	1	92	92	0.00	5.3	Pass
	2	186	185	- 0.54	5.3	Pass
AST, IU/L	1	104	106	1.9	5.0	Pass
	2	231	234	1.3	5.0	Pass
ALP, IU/L	1	161	161	0.00	6.0	Pass
	2	324	322	- 0.62	6.0	Pass
TBIL, μmol/L	1	47	47	1.3	5.0	Pass
	2	107	105	- 2.4	5.0	Pass
QC - quality control. TEa - total allowable error. K - potassium. Na - sodium. Cl - chloride. Ca - calcium. Crea - creatinine. Glu - glucose. TP - total protein. Alb - albumin. Chol - cholesterol. TG - triglyceride. ALT - alanine aminotransferase. AST - aspartate aminotransferase. ALP - alkaline phosphatase. TBIL - total bilirubin.						

**Table 2:** Comparison of quality control results of clinical immune tests before and after the reverse osmosis membrane replacement

**Parameter**	**QC level**	**QC results** **before replacement**	**QC results** **after replacement**	**Bias** **(%)**	**1/3 TEa** **(%)**	**Conclusion**
AFP, μg/L	1	9.2	9.0	- 2.2	8.3	Pass
	2	211.0	209.0	- 1.0	8.3	Pass
CEA, μg/L	1	2.4	2.3	- 4.2	8.3	Pass
	2	54.8	53.0	- 3.3	8.3	Pass
CA19-9, KU/L	1	25.1	24.6	- 2.0	8.3	Pass
	2	214.0	214.0	0.00	8.3	Pass
CA125, KU/L	1	22.0	21.8	- 0.91	8.3	Pass
	2	178.0	178.0	0.00	8.3	Pass
CA15-3, KU/L	1	21.8	21.1	- 3.2	8.3	Pass
	2	107.0	108.0	0.93	8.3	Pass
fT3, pmol/L	1	3.7	3.7	0.55	8.3	Pass
	2	21.9	21.6	- 1.4	8.3	Pass
fT4, pmol/L	1	14.6	15.2	4.1	8.3	Pass
	2	67.5	67.5	0.00	8.3	Pass
TSH, μIU/ml	1	0.49	0.47	- 4.1	8.3	Pass
	2	34.80	33.80	- 2.9	8.3	Pass
QC - quality control. TEa - total allowable error. AFP - alpha-fetoprotein. CEA - carcinoembryonic antigen. CA19-9 - carbohydrate antigen 19-9. CA125 - carbohydrate antigen 125. CA15-3 - carbohydrate antigen 15-3. fT3 - free triiodothyronine. fT4 - free thyroxine. TSH - thyroid-stimulating hormone.						

### Effect of RO membrane replacement on TG test results of serum samples

**Figure 2:**
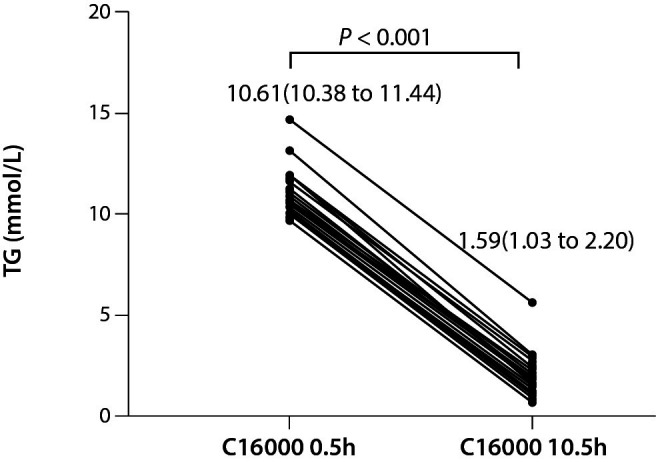
The comparison of triglyceride (TG) results of clinical serum samples on C16000 at different time points after reverse osmosis (RO) membrane replacement. The concentration of TG in 29 clinical serum samples were measured on C16000 at 0.5h and 10.5h after RO membrane replacement, and TG results at two time points were compared. Results were expressed as median and interquartile range and were analyzed by Wilcoxon test.

**Figure 3:**
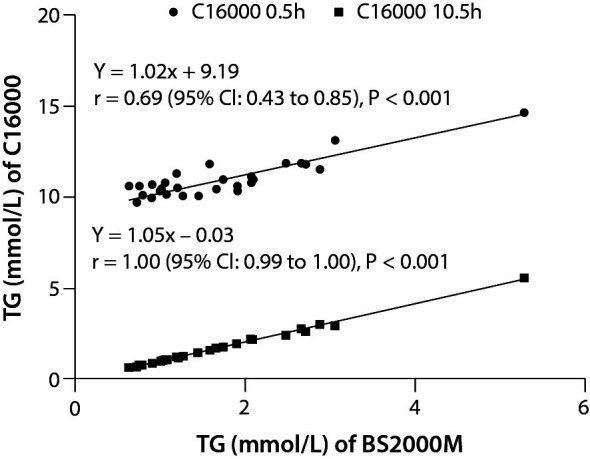
The Passing-Bablok regression and correlation analysis of triglyceride (TG) results of clinical serum samples between C16000 and BS2000M. Triglyceride of 29 clinical serum samples were measured on C16000 at 0.5h and 10.5h after reverse osmosis (RO) membrane replacement, and, meanwhile, were measured on BS2000M. Results of TG on the two biochemical analyzer were subjected to Passing-Bablok regression and Spearman correlation analysis. Passing-Bablok regression equation and Spearman correlation coefficient (r_s_) were shown. CI - confidence interval

The comparative analysis results showed that TG results at 0.5h after RO membrane replacement were consistently higher than those at 10.5h after RO membrane replacement on C16000 (10.6 (10.4 to 11.4) *vs* 1.6 (1.0 to 2.2), P *<* 0.001) (Figure 2). Meanwhile, Passing-Bablok and correlation analysis revealed that TG results by C16000 at 10.5h after RO membrane replacement were found to be in better agreement (slope = 1.05 (95% CI: 1.00 to 1.06), intercept = - 0.03 (95% CI: - 0.06 to 0.02)) and highly correlated (r_s_ = 1.00 (95% CI: 0.99 to 1.00), P < 0.001) with TG results by BS2000M when compared to those at 0.5h after replacement (slope = 1.02 (95% CI: 0.75 to 1.29), intercept = 9.19 (95% CI: 8.71 to 9.60), r_s_ = 0.69 (95% CI: 0.43 to 0.85), P < 0.001) (Figure 3).

**Figure 4:**
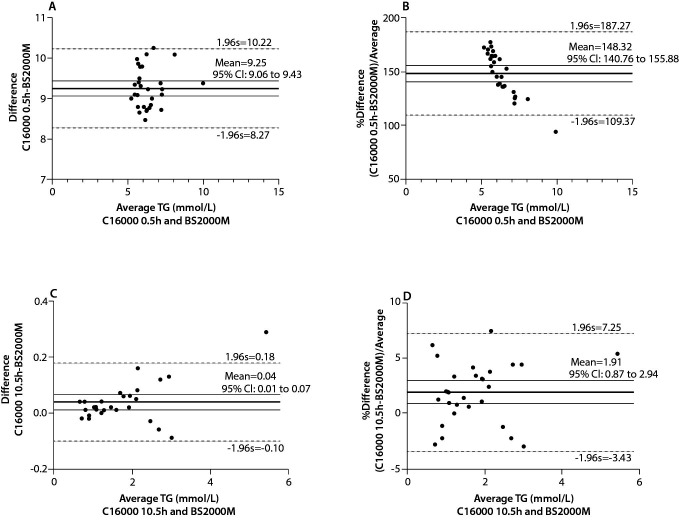
Bland-Altman plot of triglyceride (TG) results of clinical serum samples between C16000 and BS2000M. Triglyceride of 29 clinical serum samples were measured on C16000 at 0.5h and 10.5h after reverse osmosis (RO) membrane replacement, and, meanwhile, were measured on BS2000M. Bland-Altman plots were employed to analyze the relationship of TG results between C16000 and BS2000M. **(A)** Bland-Altman plots of difference of TG results between C16000 and BS2000M at 0.5h after replacement. **(B)** Bland-Altman plots of proportional difference of TG results between C16000 and BS2000M at 0.5h after replacement. **(C)** Bland-Altman plots of difference of TG results between C16000 and BS2000M at 10.5h after replacement. **(D)** Bland-Altman plots of proportional difference of TG results between C16000 and BS2000M at 10.5h after replacement. The X-axis shows the average of two groups, while the Y-axis shows the difference or proportional difference between two groups. Thick solid lines represent the mean of difference or proportional difference between two groups, thin solid lines represent the 95% confidence interval (95% CI) of mean, and dashed lines represent the 95% limits of agreement (LOA).

The Bland-Altman plots showed that there is a mean difference of 9.3 mmol/L (95% limits of agreement (95% LoA: 8.3 to 10.2) or proportional difference of 148% (95% LoA: 109% to 187%) in TG between C16000 (0.5h after RO membrane replacement) and BS2000M, while there is a mean difference of 0.04 mmol/L (95% LoA: - 0.10 to 0.18) or proportional difference of 1.9% (95% LoA: -3.4% to 7.3%) in TG between C16000 (10.5h after RO membrane replacement) and BS2000M, indicating significant greater bias of the former than the latter (Figure 4). Together, our data suggest that RO membrane replacement of water-purifying equipment may cause bias of TG test results in clinical laboratory.

### Absorbance validation of TG blank and calibrators before and after RO membrane replacement

The comparison of absorbance values of blank, calibrator level 1 (TG = 1.1 mmol/L) and calibrator level 2 (TG = 5.2 mmol/L) of TG on C16000 before and after RO membrane replacement was shown in Table 3. We found that all absorbance values increased significantly at 0.5h (blank: 0.790 ± 0.008 *vs* 0.060 ± 0.0004, P < 0.001; calibrator 1: 0.844 ± 0.012 *vs* 0.162 ± 0.0005, P < 0.001; calibrator 2: 1.204 ± 0.013 *vs* 0.561 ± 0.003, P < 0.001) and 2.5h (blank: 0.658 ± 0.029 *vs* 0.060 ± 0.0004, P < 0.001; calibrator 1: 0.743 ± 0.018 *vs* 0.162 ± 0.0005, P < 0.001; calibrator 2: 1.120 ± 0.010 *vs* 0.561 ± 0.003, P < 0.001) after RO membrane replacement compared to those before replacement. With the continuous production and drainage of pure water, all absorbances gradually decreased, and returned to normal values at 10.5h (blank: 0.061 ± 0.0005 *vs* 0.060 ± 0.0004, > 0.999; calibrator 1: 0.164 ± 0.001 *vs* 0.162 ± 0.0005, 0.997; calibrator 2: 0.561 ± 0.003 *vs* 0.561 ± 0.003, > 0.999) after RO membrane replacement compared to those before replacement.

**Table 3:** Absorbance variations of blank and triglyceride calibrators before and after RO membrane replacement

**Sample**	**Before**	**After**			P*	**P^†^**	**P^‡^**
		**0.5h**	**2.5h**	**10.5h**			
Blank	0.060 ± 0.0004	0.790 ± 0.008	0.658 ± 0.029	0.061 ± 0.0005	< 0.001	< 0.001	> 0.999
Calibrator1	0.162 ± 0.0005	0.844 ± 0.012	0.743 ± 0.018	0.164 ± 0.001	< 0.001	< 0.001	0.997
Calibrator2	0.561 ± 0.003	1.204 ± 0.013	1.120 ± 0.010	0.561 ± 0.003	< 0.001	< 0.001	> 0.999
Data are expressed as mean and standard deviation. Data were compared using one-way ANOVA followed by Dunnett’s *post-hoc* test. P*: 0.5h *vs* Before. P^†^: 2.5h *vs* Before. P^‡^: 10.5h *vs* Before. P < 0.05 was considered statistically significant.							

## Discussion

We demonstrated that RO membrane replacement could interfere TG test while had no significant effects on other laboratory tests involved in this study. Our data provide important reference for management of laboratory test interference (especially TG) caused by RO membrane replacement.

The resistivity is an important parameter for evaluating water-purifying equipment condition as well as water quality. In this study, we found that the water resistivity met required standard at all time points. After the replacement, the resistivity increased slightly and then stabilized continuously, indicating that new-installed RO membrane needs some time to reach its optimal state.

During the replacement of RO membrane, we performed QC measurement to identify the affected tests. Surprisingly, within a short period after RO membrane replacement, all QC results of clinical chemistry and immune tests passed except TG that showed serious positive bias at both QC levels. Consistently, we confirmed that the situation occurred in TG test of clinical serum samples. Moreover, RO membrane replacement could lead to worse agreement and correlation of TG test results with reference biochemical analyzer. With the continuous production and drainage of pure water (10.5h), TG test results returned to normal and had well agreement and correlation with reference biochemical analyzer. Together, these findings suggest that RO membrane replacement of water-purifying equipment interferes with TG test in clinical laboratory, leading to significant bias and instability of TG results.

The results showed that the absorbance of both blank and calibrators increased significantly at 0.5h and 2.5h after replacement, indicating the presence of interfering substances, such as glycerol, in pure water within a short period after RO membrane replacement. With the continuous water production and drainage, the absorbance returned to normal at 10.5 hours after replacement compared to that before replacement, indicating gradual elimination of interfering substances in pure water. Thus, our data suggest that RO membrane replacement of water-purifying equipment could lead to significant positive bias of TG results *via* increasing absorbance values of biochemical reaction of TG test. Indeed, we confirmed consistently that this interference indeed occurred in TG testing of clinical samples. The duration of the effect is between 2.5h and 10.5h in our system.

By employing QC measurement and clinical samples validation, we demonstrated that the replacement of key component RO membrane of water-purifying equipment could interfere TG test while have no significant effects on other laboratory tests studied. Further analysis found that RO membrane replacement may be accompanied by interfering substances in pure water to affect TG testing reactions, which requires laboratory attention. But this contamination can be avoided by continuously flushing the RO system.

There are some limitations in the study. Firstly, although RO membrane preservation solution contains glycerol, further experiments were not conducted to identify the interfering substance of TG measurement to confirm whether it was glycerol. Secondly, the testing intervals were relatively long (2.5h-10.5h), which made it difficult to know the exact time and water volume required for laboratory test results returning to normal.

In conclusion, the replacement of RO membranes may interfere with laboratory testing. It is not enough that only water resistivity meets required when important component like RO membrane is replaced in water-purifying equipment. The laboratory can eliminate the interference by flushing the RO system. However, the specific flushing time and water volume need to be determined based on the actual situation of different laboratory. Additionally, it is recommended to perform full QC checks prior to releasing any test results. In this way, the quality of laboratory testing can be guaranteed.

## Data Availability

The data generated and analyzed are available from the corresponding author on request.
